# Everybody's Column

**Published:** 1891-02-14

**Authors:** 


### Everybody's Column.

Smoke abatement being a question of heartrending moment to the Londoner, it may interest him to hear of a new stove which is announced by the "Génie Civil" to fulfil all the conditions necessary to render combustion of the fuel employed in it perfect.

The recent prolonged frost has much to answer for. Not satisfied with bursting our pipes, and generally increasing our domestic expenditure, it has caused widespread ruin in the homes of the oysters at Whitstable, where in many places when the sea was frozen over, the frost caused the oysters to swell and open their shells with fatal results.

How the announcement of "Great slaughter sale of babies' buggies" in the windows of a shop in Piccadilly or Regent Street would harrow the feelings of the fond British mother! This is the playful but terse way in which our friends in America delight to express themselves. In our roundabout way we should say, "Great sale of perambulators at immense sacrifice."

It is reported that the attendance at the first general meeting of the Society for Preserving Memorials of the Dead was small. We can hardly imagine how anything else could be expected of a society with such a lugubrious title. Even the humorous suggestion of some unknown member that an exhibition of photographs of monuments that wanted repairing should be held, with a view to making the society more widely known was treated, it would appear, with a funereal seriousness which cannot have added to the hilarity of the proceedings.

In some respects the rat is a fortunate and much-to-be-envied animal. He is free from many of the ills to which our human flesh is heir, and he can steal an egg without a blush and enjoy it without a consciousness of having done evil, accomplishments betokening a mental superiority to which the law-abiding citizen is unable to attain. But his immunity from diphtheria and other human diseases, and from anthrax, the nature of which is better known to the farmer than the ordinary layman, seems to be a mixed blessing. Science, we are informed, intends to utilise her knowledge to the detriment of the rat, and, we hope, to the benefit of mankind.

The grave question of the depopulation of France has recently been the subject of an important discussion before the Académie de Médecine. The diminution in the birth-rate, which in 1867 was regarded by many members of the Academy as a benefit to the country, is to-day looked upon as a national danger. Though Professors Le Fort and Brouardel differ as to the cause of the diminution of births, the conclusions at which they arrive are somewhat similar. Professor Le Fort, having shown that the slight increase in the French population is not due to an excessive death-rate in comparison with that of other countries, does not hesitate to lay the blame of this diminution on the code civil. The evil, he considers, originated with the introduction of this code, and will last so long as it remains unaltered. The inducements which it holds out to parents to keep down the numbers of the family, owing to the subdivision of property, are answerable also for the lowering of the moral standard and material power of the nation. Professor Brouardel also looks to an alteration of the laws as a remedy, but his view of the cause is a very different one. He maintains that every year 30,000 French people, the greater number of whom are children and men of less than thirty years of age, succumb to preventable diseases, such as typhoid and small-pox, which should be kept under by fresh sanitary legislation making vaccination compulsory.

